# Book Review

**Published:** 1917-07

**Authors:** 


**Manual of Surgery.** By Alexis Thomson and Alexander Miles. Fifth Edition. Vol. I. Pp. xix, 801. Vol. II. Pp. xvi, 948. London: Oxford Medical Publications, 1915. We have so frequently had occasion to speak in favourable terms of this manual that it seems superfluous to make any further comment in this direction. Another edition speaks for itself. In this fifth edition, as in all previous ones, various sections have been almost entirely rewritten, and new material added to bring the work up to date. After the fourth edition a companion volume was added dealing with operative surgery, and the deletion of this part from the other volume has enabled much new material to be added without appreciable increase in their size. Such increase as there is in the second volume is due largely to an addition in the number of plates. The space given to them is well expended.

**Surgical Contributions.** By Rutherford Morison. Vol. I.—General Surgery. Vol. II.—Abdominal Surgery. Messrs. J. Wright & Co.—These volumes are a collection of the total surgical contributions of their distinguished author to various journals from 1881 to 1916. They are charmingly written, and full of ripe surgical wisdom and experience. The contents are very various—technical articles on diagnosis and treatment, reviews of books, statistics of cases, accounts of trips to American clinics, and tables of aphorisms all find a place. The illustrations are numerous and excellent.

**The Mortality from Cancer throughout the World.**—By Frederick L. Hoffman, LL.D. Pp. 826. The Prudential Press, New Jersey, 1915.—This is an exhaustive work containing statistics bearing on all aspects of cancer, its mortality, its relations to other diseases, its possible causes, even on cancer houses, and cancer villages. The amount of labour expended in compiling the statistics must have been enormous, and they are no doubt of great value in connection with various theories about cancer. Cancer as a problem in life insurance is very carefully considered. Indeed, the book is written by the Statistician to the Prudential Insurance Co. of America.

**Cleft Palate and Hare Lip.** By Sir W. Arbuthnot Lane, Bart., M.S., F.R.C.S. Third Edition. Pp. 102. London: Adlard and Son, 1916. Price, 10s. net.—In reviewing the new edition of Sir W. Arbuthnot Lane's well-known book, it is not necessary to consider the question whether operation for cleft palate should be undertaken in infancy or deferred until early childhood, as the matter has been discussed at great length, and opinion on the subject is much divided. All Sir Arbuthnot Lane's methods of operating are clearly described, and illustrated by many helpful drawings. But to this new edition is added a very valuable chapter by Mr. Cortlandt MacMahon, who is instructor of speech defects at St. Bartholomew's Hospital, on the treatment of the speech defects which are associated with the cleft palate, and which require some careful and prolonged correction, after the cleft in the palate has been closed. This new edition has also a chapter by Mr. Maude James, on the means by which the teeth are restored to their normal position and function. Mr. Maude James has a large experience of the treatment of such cases at Great Ormond Street Children's Hospital.

**A Code of Rules for the Prevention of Infectious Diseases in Schools.** Issued by the Medical Officer's of Schools Association. Seventh Edition. Pp. 32. London: J. &. A. Churchill, 1915. (Price, 1s. net.)—This useful booklet has now reached its seventh edition since thirty years ago when the first edition was published. It is most essential that there should be a standard of uniformity in the various schools of the country, and this code has become quite the standard to be consulted on occasion. It has been carefully revised, but little alteration has been needful, excepting as regards Diphtheria, the rules for which disease have been altered so as to eliminite as far as possible the spread of this disease by "carrier." Now that cerebro-spinal fever has been by no means rare in schools and camps, we think it time that Rules for the detection and treatment of the carrier of the meningococcus should be included in the next edition.

**Index-Catalogue of the Library of the Surgeon-General's Office, United States Army. Authors and Subjects.** Second series. Vol. XXI., Waterworth—Vysman. Washington: Government Printing Office, 1916. Pp. 457 and 233.—This volume brings the present series to a close, and the librarian presents an interesting historical summary of the inception of the work and the growth of the library to which it refers. To Dr. John S. Billings, the creator of the Index-Catalogue and to Dr. Robert Fletcher most of the credit for the work is due, and they and their collaborators have placed the medical profession under a deep debt of gratitude. The extent of their labours is shown in part by the statement that the library now contains a total of 224,552 volumes and 337,120 pamphlets, in all 561,642 items (volumes and pamphlets). The wideness of their research is also shown by the fact that the list of abbreviations of titles of medical periodicals employed in the second series of the Index-Catalogue occupies 233 pages of the present volume. The work is indispensable, and the publication of the third series will be welcomed by all interested in medical literature.

**A War Cookery Book for the Sick and Wounded.** By Jessie M. Laurie. Pp. 60. London: T. Werner Laurie Ltd. N.D. 6d. net.—The suggestions in this booklet are invaluable; they are simple and not too extravagant, also their variety is a great asset, as it is often so difficult to think of appetising dishes which may be eaten by invalids. At the same time, we think that in a large military hospital the recipes would not come under the War Office regulation diet. A little more explanation with regard to Benger's Food, and a note to say that it should be left standing for fifteen minutes for digestive processes to go on, and then boiled, would be an advantage for the Red Cross Nurse to know. All ladies who are tending the sick and wounded may be recommended to supply themselves at once with this cookery book.

